# Incontinence and cognitive function in Chinese old adults: multiple mediating effects of social participation and depressive symptoms

**DOI:** 10.3389/fpubh.2026.1858662

**Published:** 2026-07-06

**Authors:** Xinlin Li, Tingyao Nie, Lu Lin, Anling Sun, Jun Feng, Penghui Xia

**Affiliations:** 1 Department of Geriatrics, The First Affiliated Hospital of Hunan University of Chinese Medicine, Changsha, Hunan, China; 2 Xiangya School of Nursing, Central South University, Changsha, Hunan, China

**Keywords:** cognitive function, depressive symptoms, incontinence, multiple mediation effect, social participation

## Abstract

**Background:**

China has entered a stage of deep aging. The cognitive function level of the old adults is not only an important indicator to measure their health status and quality of life, but also a key factor reflecting their social adaptability and overall physical and mental state. Against this backdrop, incontinence, as a common health issue among the old adults, not only affects their daily lives but may also further impair cognitive functions through multiple psychological and social mechanisms. Therefore, in order to gain a more comprehensive understanding of the potential pathways of interaction between incontinence and cognitive function in the old adults, this study aims to explore the multiple mediating effects of social participation and depressive symptoms in the relationship between the two, providing theoretical basis and practical reference for promoting cognitive health and enhancing the overall well-being of the old adults.

**Methods:**

According to the 2018 China Longitudinal Health and Longevity Survey (CLHLS) dataset, a total of 9,299 Chinese old adults aged 65 and above were selected. Firstly, the basic socio-demographic characteristics of the sample population were described. Secondly, a Spearman correlation analysis was conducted to examine the associations among incontinence, social participation, depressive symptoms and cognitive function in the old adults. Finally, sequential multiple mediating analysis was conducted using SPSS Macro PROCESS 4.0 to evaluate the potential mediating role of social participation and depressive symptoms.

**Results:**

Incontinence, social participation, depressive symptoms and cognitive function were significantly correlated (*p* < 0.01). Incontinence is not only directly related to the cognitive function of the old adults (effect = −4.1964; but also has a standard error of 0.2219. 95% confidence interval: LL = −4.6314, UL = −3.7615), and the independent mediating effect of social participation (effect = −0.2256; standard error = 0.0271; 95% confidence interval: LL = −0.2795, UL = −0.1722), independent mediating effect of depressive symptoms (effect = −0.0856; standard error = 0.0285; 95% confidence interval: LL = −0.1438, UL = −0.0328), the chain mediating effect between social participation and depressive symptoms (effect = −0.0096; standard error = 0.0022;95% confidence interval: LL = −0.0142, UL = -0.0057) was associated with major cognitive function.

**Conclusion:**

This study suggested that social participation and depressive symptoms may be involved in the association between incontinence and cognitive function among older adults in China. These findings highlight the importance of paying attention to social participation and depressive symptoms in older adults with incontinence, which may help inform strategies for cognitive health promotion.

## Introduction

China is experiencing an unprecedentedly severe population aging. Currently, 12.0% of the population (173 million) is aged 65 and above, and this is projected to reach 26.1% (366 million) by 2050 (1). With increasing life expectancy and declining fertility rates (2, 3), this trend is expected to intensify.

Cognitive impairment is a condition closely associated with aging. As the population ages, its prevalence is increasing (4). Among individuals aged 65 and older, the prevalence of mild cognitive impairment is estimated to range from 3 to 22% (5), and in China, from 9.7 to 23.3% (6, 7). Studies suggest that declines in subjective (mild) cognitive function may serve as an early indicator of dementia and can substantially reduce health-related quality of life in older adults (8). Moreover, cognitive impairment can lead to functional loss, placing additional burdens on families and society (9). Therefore, effectively understanding and identifying the factors influencing cognitive function is particularly important.

### Incontinence and cognitive function

Incontinence primarily includes urinary and fecal incontinence. Urinary incontinence is defined by the International Continence Society as the involuntary leakage of urine (10), while fecal incontinence refers to the involuntary passage of solid or liquid stool (11). Research indicates that the prevalence of incontinence increases with age (12). A meta-analysis reported that urinary incontinence is most prevalent among older adults in Asia, reaching 45.1% (13). In China, a survey found that 46.8% of rural older adults aged 65 and above experience urinary incontinence (14). Incontinence not only affects physical health but also impacts psychological well-being and subjective life satisfaction (15). Moreover, fecal incontinence is closely associated with mortality (16). Existing studies have shown that incontinence is linked to cognitive decline in older adults (17, 18). Notably, the potential pathways between incontinence and cognitive function have not yet been explored. Therefore, this study aims to investigate the underlying mechanisms linking incontinence and cognitive function among Chinese older adults.

### Incontinence, cognitive function, social participation, and depressive symptoms

Beyond the direct impact of incontinence on cognitive function in older adults, exploring the underlying mechanisms of this pathway is also of interest. Engagement in activities can enhance cognitive reserve, which is closely associated with cognitive function in older adults (19). The relationship between social participation and cognitive function has been widely documented (20–22). In addition, incontinence has been found to affect social participation by limiting physical activity and impacting mental health in older adults (23). Given these associations, older adults with incontinence may exhibit lower levels of social participation, which in turn could further reduce cognitive function. Therefore, one of the objectives of this study is to examine whether social participation mediates the relationship between incontinence and cognitive function in older adults.

Previous research supports the notion that depressive symptoms may mediate the relationship between incontinence and cognitive function. Depression is one of the most common mental disorders worldwide and is particularly prevalent among older adults (24). Substantial evidence indicates that social engagement can effectively reduce the risk of depressive symptoms in this population (25–27). A longitudinal study in South Korea on middle-aged and older women with urinary incontinence found that more severe incontinence was associated with increased depressive symptoms (28). Moreover, the association between depressive symptoms and cognitive function has been widely established (29–31). Therefore, we hypothesize that social participation may influence depressive symptoms, which in turn could serve as a potential mediator in the relationship between incontinence and cognitive function in older adults.

The stress process model suggests that chronic health conditions such as incontinence can act as persistent stressors, affecting not only an individual’s emotional state but also potentially impairing cognitive processing through prolonged psychological burden (32). Concurrently, cognitive reserve theory posits that positive social interactions and environmental stimulation can enhance cognitive reserve in older adults, providing a protective effect against adverse health factors, whereas restricted social participation reduces cognitive stimulation, increasing vulnerability to cognitive decline (33). In addition, socioemotional selectivity theory emphasizes that older adults’ emotion regulation relies heavily on social resources and emotional support networks; reduced social participation is often accompanied by increased loneliness and helplessness, elevating the risk of depression, which itself is a recognized psychological pathway to cognitive decline (34). Collectively, psychosocial resources and emotional states constitute key pathways linking health status to cognitive performance. Accordingly, this study conceptualizes social participation and depressive symptoms as sequential mediators in the pathway from incontinence to cognitive function, aiming to elucidate the potential underlying mechanisms.

### The current study

Based on the above empirical findings and theoretical considerations, this study aims to investigate the potential mechanisms linking incontinence and cognitive function in older adults. Specifically, it examines a sequential multiple mediation model in which social participation and depressive symptoms serve as mediating pathways. The following hypotheses are proposed: (1) incontinence is negatively associated with cognitive function in older adults; (2) social participation and depressive symptoms independently mediate the relationship between incontinence and cognitive function; and (3) social participation and depressive symptoms may be involved in a sequential mediating pathway linking incontinence to cognitive function, with incontinence being associated with lower social participation, more depressive symptoms, and poorer cognitive function.

## Methods and measurements

### Data source and sample

**Figure 1:**
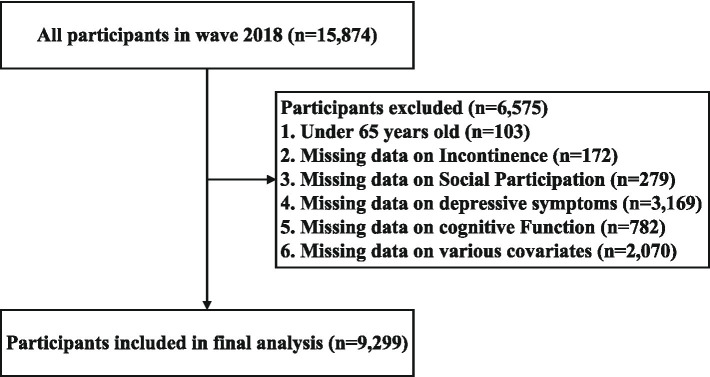
Filter flowchart.

This study utilized data from the 2018 wave of the Chinese Longitudinal Healthy Longevity Survey (CLHLS). The survey, organized by the Center for Healthy Aging and Development Studies at Peking University, covers older adults aged 65 and above across 23 provinces in China and employs a multistage, non-proportional, and targeted sampling design. Its data quality has been widely recognized in both domestic and international research. In the 2018 round, the survey collected health information from 15,874 older adults, including measures of incontinence, social participation, depressive symptoms, and cognitive function. As this study focused exclusively on individuals aged 65 and above, and some participants with limited mobility did not complete the questionnaire, these cases were excluded. Ultimately, 9,299 valid samples were retained. The sample screening process is shown in Figure 1. The CLHLS study was approved by the research ethics committees of Duke University and Peking University (IRB00001052–13074). The procedures used in this study adhere to the tenets of the Declaration of Helsinki. Informed consent was obtained from all individual participants included in the study.

### Measurements

#### Incontinence

Older adults’ incontinence status (35) was assessed using the question “Are you able to control your bowel movements and urination?” The response options included “able to control both bowel movements and urination,” “occasional incontinence,” and “unable to control or requiring catheter assistance,” which were assigned scores of 1, 2, and 3, respectively. Higher scores indicated more severe incontinence.

#### Cognitive function

Cognitive function in the CLHLS was assessed using a standardized cognitive screening instrument adapted for the Chinese older population (31). The questionnaire consists of 24 items, assessing orientation, counting food in 1 min, attention, calculation, drawing, recall and language skills. The score range for counting food items per minute is from 0 to 7 points. One point is awarded for each correctly counted food item, with a maximum of 7 points. For other questions, 1 is correct and 0 is incorrect. The score range is from 0 to 30. The higher the score, the better the cognitive function.

#### Social participation

Social participation was assessed using six items from the CLHLS, including outdoor activities, playing cards or mahjong, and participating in organized social activities (15). Outdoor activities included Tai Chi, square dancing, visiting others, socializing with friends, and other outdoor activities. Each item was originally rated on a five-point scale according to participation frequency: 1 = almost every day, 2 = at least once a week, 3 = at least once a month, 4 = sometimes, and 5 = not at all. To facilitate interpretation, all items were reverse-coded before analysis, so that higher scores represented more frequent participation. The total score was calculated by summing the reverse-coded item scores, with higher scores indicating a higher level of social participation.

#### Depressive symptoms

Depressive symptoms were measured using the 10-item Center for Epidemiologic Studies Depression Scale (CES-D-10) (36). Each item was rated on a five-point scale: 1 = always, 2 = often, 3 = sometimes, 4 = rarely, and 5 = never. For negatively worded items, the original coding was retained, whereas positively worded items were reverse-coded so that all items were scored in the same direction. The total score was calculated by summing the scores of the 10 items, with a possible range of 10 to 50. In this study, lower CES-D-10 scores indicated more severe depressive symptoms, whereas higher scores indicated fewer depressive symptoms.

### Statistical analysis

All statistical analyses were performed using IBM SPSS Statistics version 27.0. Descriptive statistics were used to summarize the general characteristics of the study population. Continuous variables conforming to a normal distribution were expressed as mean ± standard deviation, whereas skewed continuous variables were presented as median and interquartile range. Categorical variables were reported as frequencies and percentages. Considering the measurement characteristics of the study variables and the purpose of examining monotonic associations, Spearman correlation analysis was used to examine the relationships among incontinence, social participation, depressive symptoms, and cognitive function. Before the mediation analysis, potential confounding factors were screened using univariate linear regression analyses, with cognitive function as the dependent variable and each demographic and clinical characteristic as the independent variable. Variables significantly associated with cognitive function, including age, gender, marital status, years of schooling, self-assessment of health, and subjective economic condition, were included as covariates in the final mediation model. To test the multiple mediating effects between incontinence and cognitive function, the SPSS Macro PROCESS 4.0 developed by Hayes (37) was applied using Model 6. The indirect effects were estimated using the bootstrap method with 5,000 resamples, and 95% confidence intervals were calculated. A mediating effect was considered statistically significant when the 95% confidence interval did not include zero. A two-sided *p* value < 0.05 was considered statistically significant.

### Common method biases

This study employed Harman’s single-factor test to detect common method deviations (38). The results without rotating the factors show that the eigenvalues of 14 factors are greater than 1, indicating that there are multiple factors behind the data. The variance explained by the first factor is 8.9%, which is lower than the threshold of 40%. The above analysis indicates that there is no serious common methodological bias in our research.

## Results

### Characteristics of samples

Table 1 shows the demographic characteristics of the participants. In our study, the average age of the old adults was 83.20 ± 11.29 years, and the average school age was 3.75 ± 4.41 years. Among the total respondents, 4,258 (45.8%) were male and 5,041 (54.2%) were female. More than 40% of the old adults live in rural areas, while the proportion of the old adults in urban areas (32.5%) and cities (26.0%) is not much different. The spouses of the majority of old adults in China are either alive and living together (45.2%) or have passed away (51.7%). Most old adults (80.0%) choose to live with their families to enjoy their old age together. At present, the majority of the old adults do not smoke or drink alcohol, accounting for 84.1 and 84.8%, respectively. The average economic situation of the majority of respondents was 70.1%. As for self-assessment of health, the majority of the old adults consider their health condition to be average (38.5%) or good (35.5%), a small number of the old adults think their health condition is very good (12.6%) or poor (12.4%), and only a very small proportion of the old adults consider their health condition to be very poor (1.0%).

**Table 1:** The characteristics of the sample (*N* = 9,299).

Variables	Category	*N*	Mean ± SD/Percentage (%)
Age		9,299	83.20 ± 11.29
Years of schooling		9,299	3.75 ± 4.41
Gender	Male	4,258	45.8
	Female	5,041	54.2
Current residence	City	2,420	26.0
	Town	3,024	32.5
	Rural	3,855	41.5
Marital status	Currently Married and Living with Spouse	4,206	45.2
	separated	173	1.9
	Divorced	32	0.3
	Widowed	4,812	51.7
	Never Married	76	0.8
Co-residence of respondent	With Household Members	7,443	80.0
	Alone	1,530	16.5
	In an Institution	326	3.5
Smoking	Yes	1,481	15.9
	No	7,818	84.1
Drinking	Yes	1,409	15.2
	No	7,890	84.8
Subjective economic condition	Very rich	269	2.9
	rich	1,612	17.3
	So so	6,523	70.1
	poor	785	8.4
	Very poor	110	1.2
Self-assessment of health	Very good	1,170	12.6
	Good	3,305	35.5
	So so	2,579	38.5
	bad	1,151	12.4
	Very bad	94	1.0

SD, Standard Deviation.

Taking cognitive function as the dependent variable and each covariate as the independent variable, a multiple linear regression analysis was conducted. The results showed that age, gender, marital status, years of schooling, self-assessment of health, and subjective economic condition were significantly associated with cognitive function among older adults (*p* < 0.05) (Table 2).

**Table 2:** Multiple linear regression analysis of cognitive function in the old adults (*N* = 9,299).

Variables	*β*	SE	*t*	*p*
Age	−0.235	0.006	−42.363	**<0.001**
Years of schooling	0.164	0.014	11.550	**<0.001**
Gender	−0.912	0.119	−7.676	**<0.001**
Current residence	−0.135	0.069	−1.957	0.050
Marital status	−0.127	0.044	−2.869	**0.004**
Co-residence of the respondent	0.187	0.108	1.723	0.085
Smoking	0.099	0.151	0.657	0.511
Drinking	0.029	0.151	−0.192	0.848
Subjective economic condition	−0.406	0.084	−4.864	**<0.001**
Self-assessment of health	−0.833	0.058	−14.387	**<0.001**

The bolded items represent the specific statistical effect indicators.

### Correlation of incontinence, cognitive function, social participation, and depressive symptoms

Table 3 lists the correlations among the examined variables. The relevant analysis results were consistent with our expected assumptions. All analysis results were statistically significant at the *p* < 0.01 (two-tailed) level. Firstly, incontinence was significantly negatively correlated with cognitive function (*r* = −0.191) and depressive symptoms (*r* = −0.101), and significantly positively correlated with social participation (*r* = 0.163). Secondly, cognitive function was significantly negatively correlated with social participation (*r* = −0.382) and significantly positively correlated with depressive symptoms (*r* = 0.220). Finally, social participation was significantly negatively correlated with depressive symptoms (*r* = −0.165).

**Table 3:** The correlation between incontinence, cognitive function, social participation and depressive symptoms.

Variables	Mean ± SD	Incontinence	Cognitive function	Social participation	Depressive symptoms
Incontinence	1.04 ± 0.224	1			
Cognitive function	25.52 ± 5.87	−0.191^*^	1		
Social participation	25.82 ± 3.91	0.163^*^	−0.382^*^	1	
Depressive symptoms	37.79 ± 6.13	−0.101^*^	0.220^*^	−0.165^*^	1

All values in the table represent Spearman’s rank correlation coefficients (r); *p < 0.01 (two-tailed).

### Mediation analysis of social participation and depressive symptoms

We used the bootstrap method to explore the role of social participation and depressive symptoms in the relationship between incontinence and cognitive function among the respondents. The control subjects included age, gender, marital status, years of education, self-rated health and subjective economic status (see Table 4). The results showed that incontinence was positively correlated with social participation (*β* = 1.2611, *p* < 0.001) and negatively correlated with depressive symptoms (*β* = −0.9742, *p* < 0.001). Social participation was negatively correlated with depressive symptoms (*β* = −0.0870, *p* < 0.001). We found that, after adjusting for all other variables, social participation (*β* = −0.1789, *p* < 0.01) and depressive symptoms (*β* = 0.0878, *p* < 0.001) were significantly associated with cognitive function. When social participation and depressive symptoms were included in the regression analysis, the relationship between incontinence and cognitive function remained significant (*β* = −4.1964, *p* < 0.01).

**Table 4:** A sequential multiple mediation model between incontinence and cognitive function in older adults.

Predictors	Social participation			Depressive symptoms			Cognitive function		
	*β*	SE	*t*	*β*	SE	*t*	*β*	SE	*t*
Age	0.1002	0.0040	24.7653^***^	−0.0098	0.0062	−1.5761	−0.1980	0.0055	−36.1505^***^
Years of schooling	−0.1397	0.0096	−14.5420^***^	0.0541	0.0145	3.7291^***^	0.1568	0.0127	12.3038^***^
Marital status	−0.0218	0.0305	−0.7134	−0.3018	0.0456	−6.6156^***^	−0.0687	0.0401	−1.7117
gender	−0.1394	0.0809	−1.7240	−0.5128	0.1208	−4.2438^***^	−0.8344	0.1062	−7.8585^***^
Self-assessment of health	0.4159	0.0426	9.7646^***^	−2.7288	0.0639	−42.6731^***^	−0.4002	0.0614	−6.5187^***^
Subjective economic condition	0.4167	0.0613	6.8003^***^	−1.3095	0.0918	−14.2689^***^	−0.1802	0.0814	−2.2132^*^
Incontinence	1.2611	0.1686	7.4817^***^	−0.9742	0.2526	−3.8574^***^	−4.1964	0.2219	−18.9124^***^
Social participation				−0.0870	0.0155	−5.6131^***^	−0.1789	0.0136	−13.1254^***^
Depressive symptoms							0.0878	0.0091	9.6422^***^
*R* ^2^	0.1684			0.2468			0.3669		
*F*	268.8419			380.5875			598.0502		

*β*, standardized coefficients; SE, Standard Error, *p < 0.05, **p < 0.01, ***p < 0.001.

**Figure 2:**
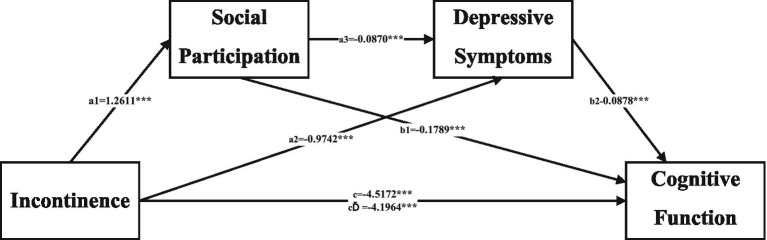
Continuous mediation model of incontinence, cognitive function, social participation and depressive symptoms. The path coefficients have been shown in the standardized regression coefficients. *p < 0.05, **p < 0.01, ***p < 0.001.

The results of the sequential mediation analysis of social participation and depressive symptoms between incontinence and cognitive function are shown in Figure 2 and Table 5. The overall impact of incontinence on the main cognitive function is significant (*p* < 0.01); that is, more severe incontinence was significantly associated with poorer cognitive function among older adults.

**Table 5:** Hypothesized continuous mediation model: the association between social participation and depressive symptoms in incontinence and cognitive function.

Pathway	Effect	SE	BootLLCI	BootULCI
Total effect (c)	−4.5172	0.2243	−4.9570	−4.0774
Direct effect (c’)	−4.1964	0.2219	−4.6314	−3.7615
a1	1.2611	0.1686	0.9307	1.5916
a2	−0.9742	0.2526	−1.4692	−0.4791
a3	−0.0870	0.0155	−0.1174	−0.0566
b1	−0.1789	0.0136	−0.2056	−0.1522
b2	0.0878	0.0091	0.0700	0.1057
Indirect effects				
Total indirect effects	−0.3208	0.0405	−0.4021	−0.2430
Indirect 1	−0.2256	0.0271	−0.2795	−0.1722
Indirect 2	−0.0856	0.0285	−0.1438	−0.0328
Indirect 3	−0.0096	0.0022	−0.0142	−0.0057

Indirect 1, incontinence → Social participation → Cognitive Function Indirect 2: Incontinence → Depressive symptoms → cognitive function; Indirect 3: Incontinence → Social participation → Depressive symptoms→cognitive function. BootLLCI self-help lower confidence interval, BootULCI self-help upper confidence interval, SE standard error, effect standardized regression coefficient.

In addition, these three indirect pathways are also important. Specifically, the first indirect pathway was that social participation significantly mediated the association between incontinence and cognitive function, with an effect value of −0.2256. The second indirect pathway was that depressive symptoms significantly mediated the association between incontinence and cognitive function, with an effect value of −0.0856. The third indirect pathway was that the association between incontinence and cognitive function was significantly mediated by social participation and depressive symptoms in sequence, with an effect value of −0.0096. In addition, social participation was significantly associated with depressive symptoms, with an effect size of −0.0870. The total indirect effect accounted for 7.10% of the total effect, with the indirect pathway through social participation accounting for 4.99%, the pathway through depressive symptoms accounting for 1.89%, and the sequential pathway through social participation and depressive symptoms accounting for 0.21%.

Taken together, these results suggest that social participation and depressive symptoms may play a role in the association between incontinence and cognitive function.

### Sensitivity analysis by sex

To further examine the robustness of the mediation model, sex-stratified sensitivity analyses were conducted. Among male participants, the mediation results were generally consistent with those of the full sample, with the total indirect effect and the three specific indirect pathways all reaching statistical significance. Among female participants, the indirect pathway through depressive symptoms alone was not statistically significant among female participants, as the 95% bootstrap CI included zero (effect = −0.0617, 95% bootstrap CI: −0.1304 to 0.0052). Overall, these findings suggest that the mediation model was partially robust across sex subgroups, although the depressive symptoms pathway alone may differ between male and female participants. The specific results can be found in the Appendix.

## Discussion

Based on the proposed hypotheses and the findings of this study, we aimed to explore the potential mechanisms underlying the association between incontinence and cognitive function in older adults. Specifically, we examined whether social participation and depressive symptoms served as potential mediating pathways in this relationship. Our results suggest that incontinence is negatively associated with cognitive function in older adults. In addition, we found that social participation and depressive symptoms may independently mediate the relationship between incontinence and cognitive function. Moreover, a sequential mediation model was observed, in which incontinence was associated with lower social participation, which was in turn associated with more depressive symptoms and ultimately with poorer cognitive function.

### The direct effect of incontinence on cognitive function

The present study found a significant negative association between incontinence and cognitive function, consistent with previous empirical findings (17, 18), suggesting that incontinence is a common yet often overlooked geriatric syndrome that may be related to outcomes beyond physiological discomfort. Incontinence is frequently accompanied by nocturia, urinary urgency, and fragmented sleep (39), which may interfere with deep sleep and rapid eye movement sleep—stages important for memory consolidation, neural plasticity, and cognitive processing. Prolonged disruption of sleep architecture may be associated with cognitive decline (40). Moreover, incontinence, particularly among older adults in the Chinese cultural context, is often stigmatized, which may affect healthcare-seeking behavior and be linked to poorer cognitive outcomes (41). It should also be noted that the relationship between incontinence and cognitive function may be bidirectional. Cognitive decline may be associated with impaired toileting ability, reduced awareness of bladder cues, and difficulties in planning or performing timely toileting behaviors, which may in turn be related to a higher likelihood of incontinence (42). Therefore, the observed association should be interpreted cautiously. Nevertheless, several studies have reported no association between incontinence and cognitive impairment (14, 43), underscoring the need for further longitudinal research to clarify related factors, temporal sequences, and potential causal relationships.

### The mediation effect of social participation and depressive symptoms

By applying a sequential multiple mediation model, this study further elucidated how social participation and depressive symptoms jointly constitute key pathways linking incontinence to cognitive decline. The results indicated that incontinence was significantly associated with reduced social participation in older adults, and lower levels of social participation were closely related to poorer cognitive performance. Previous research has shown that incontinence can limit older adults’ mobility and social engagement due to embarrassment, shame, or physical constraints (44). Adequate social participation not only provides emotional support and environmental stimulation but may also mitigate cognitive decline and dementia risk through mechanisms such as enhancing cognitive reserve, promoting neural plasticity, and improving cerebrovascular health (45).

Along another pathway, this study found that depressive symptoms play a key mediating role between incontinence and cognitive function. Incontinence is often accompanied by feelings of shame, helplessness, and activity limitations, which significantly increase the risk of depression (46, 47). Substantial evidence also indicates a strong negative association between depressive symptoms and cognitive impairment (48). This relationship may be related to white matter abnormalities, subcortical structural damage, and altered neural network function commonly observed in older adults with depression (49, 50). Moreover, depressive symptoms may represent an early stage of neurodegenerative diseases, such as Alzheimer’s disease and vascular dementia (51, 52).

Notably, this study is consistent with a sequential multiple mediation model involving social participation and depressive symptoms. The results suggest that social limitations associated with incontinence may be related to lower social engagement and may also be linked to more depressive symptoms through increased loneliness and negative affect, ultimately being associated with poorer cognitive function. Previous research has shown that higher levels of social participation are associated with fewer depressive symptoms (53), and fewer depressive symptoms are significantly associated with better cognitive performance (54). Thus, incontinence may be associated with a cumulative health burden among older adults, with its related difficulties extending beyond a single domain and being reflected across social, emotional, and cognitive dimensions, thereby potentially affecting overall physical and mental well-being.

Within the sociocultural context of China, the pathway linking incontinence to cognitive function through social participation and depressive symptoms carries particularly complex and salient implications. First, with the rapid aging of the population, the number of disabled and semi-disabled older adults is expanding, and incontinence is highly prevalent in this group, yet it has long been regarded as a “taboo” or private issue (55). This pronounced stigmatization in China may be associated with lower willingness among older adults to seek help, as well as lower levels of social engagement and greater social isolation, which may in turn be related to poorer cognitive function.

Second, China’s traditional family structure is rapidly shifting from extended to nuclear families, with a growing number of empty-nest and solitary older adults (56). Reduced family caregiving resources may leave older adults to face more pronounced embarrassment and psychological stress associated with incontinence on their own. Low levels of social participation and lack of emotional support may be associated with feelings of shame, neglect, and depressive symptoms, which in turn may be linked to poorer cognitive function. Meanwhile, for older adults who still rely on family members or informal caregivers, incontinence-related care needs may also place additional strain on patient–caregiver relationships (57). Therefore, the psychosocial burden of incontinence should not be viewed merely as an individual health issue, but rather as a family-centered care issue embedded within changing caregiving structures.

Furthermore, the sex-stratified sensitivity analysis indicated that the mediation findings were largely consistent between male and female participants, supporting the robustness of the main results to some extent. However, among female participants, the indirect pathway through depressive symptoms alone did not reach statistical significance. This finding suggests that the role of depressive symptoms in the association between incontinence and cognitive function may vary across sex subgroups. One possible explanation is that psychosocial experiences related to incontinence, social participation, and emotional distress may differ between older men and women (58, 59). Therefore, these sex-specific findings should be interpreted with caution and further explored in future research.

In summary, the mediation model in this study not only provides insight into the psychosocial pathways linking incontinence to cognitive function but also highlights critical vulnerabilities within China’s social structure, cultural attitudes, and healthcare system. Future interventions targeting older adults with incontinence should address both cultural factors and structural barriers, thereby providing more feasible strategies to support cognitive health among aging population.

### Practical implications

To date, studies on the potential mechanisms linking incontinence to cognitive function in older adults remain limited, particularly regarding the roles of social participation and depressive symptoms. The findings of this study have important implications for clinical nursing and public health practice. Given the identified mediating pathways and the results of the sex-stratified sensitivity analysis, interventions aimed at maintaining or improving social participation may represent a particularly important and relatively consistent strategy for older adults with incontinence. At the same time, depressive symptoms should also be considered in clinical assessment and care planning, although their specific mediating role may vary across sex subgroups. For example, community health services could develop small-scale, low-stress interactive activities tailored for older adults with mobility limitations or incontinence-related embarrassment, providing a safe, stigma-free environment for social engagement and opportunities for social interaction and cognitive stimulation. Early identification and management of depressive symptoms may also be important for supporting cognitive function, particularly when emotional distress coexists with reduced social participation. Primary care and family physician–based services can incorporate psychological screening into routine health management for older adults with incontinence and provide emotional support, counseling, or referral services to address prolonged emotional distress that may be related to poorer cognitive outcomes. In addition, public education aimed at reducing the stigma of incontinence is essential, as it may help normalize discussions and help-seeking behaviors and reduce the likelihood of social withdrawal due to shame. Future interventions may benefit from considering sex-specific psychosocial experiences and tailoring support strategies accordingly, rather than assuming that the same psychosocial pathway operates uniformly among all older adults with incontinence.

### Limitations

Several limitations of this study should be acknowledged. First, the cross-sectional design limits the ability to infer causal relationships among incontinence, social participation, depressive symptoms, and cognitive function. Future longitudinal or interventional studies are essential to clarify the temporal sequence and underlying pathways. Second, the study relied primarily on self-reported measures to assess incontinence, psychological symptoms, and social participation, which may be subject to social desirability or recall bias. Subsequent research could incorporate objective indicators to improve measurement accuracy. Third, a complete-case analysis was used for missing data on key variables, which, while simplifying data handling, may introduce selection bias and reduce the generalizability of the findings. In addition, although the CLHLS provides a large-scale and nationwide sample, its sampling design includes oversampling of the oldest-old population, which should be considered when interpreting the representativeness and generalizability of the findings. Fourth, although the current model adjusted for several key sociodemographic factors, some clinically relevant confounders were not fully accounted for, such as anticholinergic medication use, overall comorbidity burden, and functional status or activities of daily living. These factors may be associated with both incontinence and cognitive function, and their omission may have led to residual confounding. Future studies should incorporate more comprehensive clinical and functional indicators when data are available. Fifth, although sex-stratified sensitivity analyses were conducted, these analyses were exploratory in nature. The nonsignificant indirect pathway through depressive symptoms alone among female participants should therefore be interpreted cautiously, and future studies could further examine sex differences using formal interaction tests or moderated mediation models. Finally, this study did not further distinguish among different types, duration, or severity of incontinence, nor did it conduct detailed stratified analyses for comorbid chronic conditions that may affect cognition. These unaccounted sources of heterogeneity may partially limit the interpretability of the results. Future research with larger samples could explore stratified or interaction analyses based on incontinence characteristics and clinical conditions to provide a more comprehensive understanding of the mechanisms linking incontinence to cognitive function.

## Conclusion

Despite these limitations, this study provides meaningful insights into the relationships among incontinence, social participation, depressive symptoms, and cognitive function in older adults. The findings highlight the need for culturally sensitive interventions that address not only the physical symptoms of incontinence but also its psychosocial consequences. By promoting social participation and alleviating depressive symptoms, public health practitioners can help reduce the burden of incontinence and enhance the physical and mental well-being of aging population.

## Data Availability

The original contributions presented in the study are included in the article/Supplementary material, further inquiries can be directed to the corresponding authors.
