# Supplementary material for: Incontinence and cognitive function in Chinese old adults: multiple mediating effects of social participation and depressive symptoms
Source: Front Public Health. 2026 Jul 6;14:1858662. doi: 10.3389/fpubh.2026.1858662 (PMC13381464; doi:10.3389/fpubh.2026.1858662)
Supplement: Supplementary file 1 [file Table_1.DOCX]

Appendix 1. Hypothesized continuous mediation model: the association between social participation and depressive symptoms in incontinence and cognitive function(male)

| **Pathway** | **Effect** | **SE** | **BootLLCI** | **BootULCI** |
| --- | --- | --- | --- | --- |
| Total effect (c) | -4.2620 | 0.3156 | -4.8807 | -3.6433 |
| Direct effect (c’) | -3.8759 | 0.3120 | -4.4876 | -3.2642 |
| a1 | 1.6212 | 0.2714 | 1.0892 | 2.1533 |
| a2 | -1.3148 | 0.3958 | -2.0907 | -0.5389 |
| a3 | -0.1116 | 0.0223 | -0.1553 | -0.0679 |
| b1 | -0.1470 | 0.0176 | -0.1815 | -0.1125 |
| b2 | 0.0988 | 0.0121 | 0.0751 | 0.1225 |
| Indirect effects |  |  |  |  |
| Total indirect effects | -0.3861 | 0.0663 | -0.5245 | -0.2638 |
| Indirect 1 | -0.2384 | 0.0348 | -0.3091 | -0.1757 |
| Indirect 2 | -0.1299 | 0.0530 | -0.2449 | -0.0355 |
| Indirect 3 | -0.0179 | 0.0046 | -0.0277 | -0.0097 |

Appendix 2. Hypothesized continuous mediation model: the association between social participation and depressive symptoms in incontinence and cognitive function(female)

| **Pathway** | **Effect** | **SE** | **BootLLCI** | **BootULCI** |
| --- | --- | --- | --- | --- |
| Total effect (c) | -4.6361 | 0.3129 | -5.2496 | -4.0226 |
| Direct effect (c’) | -4.3708 | 0.3098 | -4.9781 | -3.7635 |
| a1 | 1.0133 | 0.2136 | 0.5945 | 1.4321 |
| a2 | -0.7702 | 0.3277 | -1.4126 | -0.1278 |
| a3 | -0.0686 | 0.0216 | -0.1109 | -0.0263 |
| b1 | -0.1954 | 0.0204 | -0.2354 | -0.1554 |
| b2 | 0.0801 | 0.0133 | 0.0540 | 0.1062 |
| Indirect effects |  |  |  |  |
| Total indirect effects | -0.2653 | 0.0525 | -0.3665 | -0.1610 |
| Indirect 1 | -0.1980 | 0.0388 | -0.2759 | -0.1238 |
| Indirect 2 | -0.0617 | 0.0339 | -0.1304 | -0.0052 |
| Indirect 3 | -0.0056 | 0.0022 | -0.0104 | -0.0019 |

Abbreviation: Indirect 1, incontinence→Social participation→Cognitive Function Indirect 2: Incontinence→Depressive symptoms→cognitive function; Indirect 3: Incontinence→Social participation→Depressive symptoms→cognitive function. BootLLCI self-help lower confidence interval, BootULCI self-help upper confidence interval, SE standard error, effect standardized regression coefficient
